# Reengineered Anti-CD4 Cys-diabody Variants for ^89^Zr-immunoPET of CD4^+^ T Cells in Immunocompetent Mice

**DOI:** 10.1007/s11307-025-02043-y

**Published:** 2025-08-07

**Authors:** Felix B. Salazar, Richard Tavaré, Arya Ökten, Maciej Kujawski, Anna M. Wu, Kirstin A. Zettlitz

**Affiliations:** 1 Crump Institute for Molecular Imaging, Department of Molecular and Medical Pharmacology, David Geffen School of Medicine, University of California, Los Angeles, CA USA; 2 Department of Immunology and Theranostics, Arthur Riggs Diabetes and Metabolism Research Institute, Beckman Research Institute, City of Hope, 1500 E. Duarte Rd, Duarte, CA 91010 USA; 3 Present Address: Regeneron Pharmaceuticals, Inc, Tarrytown, NY USA; 4 Present Address: Department of Immunobiology, Yale School of Medicine, New Haven, CT USA

**Keywords:** Antibody fragments, CDR-grafting, Zirconium-89, Aglycosylation/glycoengineering, T helper cell, Non-invasive imaging, Immune cells

## Abstract

**Purpose:**

CD4^+^ T cells (T helper and T reg) play an important role in the immune system and are influential in autoimmune diseases (e.g., rheumatoid arthritis, inflammatory bowel disease) and cancer (antitumor immunity). Non-invasive, whole-body anti-CD4 immunoPET can provide dynamic and spatial information (localization, proliferation, and migration) on CD4^+^ T cells. The cys-diabody format enables site-specific radiolabeling and rapid renal clearance, which results in high-contrast images at early time points.

**Procedures:**

In this work, an anti-CD4 cys-diabody based on the hybridoma GK1.5 was reengineered by CDR-grafting (GK1.5 FR cDb) for higher expression in mammalian cell lines. An N-glycosylation motif in the variable light chain domain framework was removed by site-directed mutagenesis, resulting in GK1.5 N80D cDb. To investigate the impact of the variable domain glycan on the *in vivo* biodistribution and pharmacokinetics, both cys-diabodies were site-specifically conjugated with deferoxamine-maleimide and radiolabeled by chelation of zirconium-89. Serial immunoPET/CT imaging was used for non-invasive, whole-body assessment of specific targeting, biodistribution, and differential clearance of the two novel anti-CD4 cys-diabodies.

**Results:**

The anti-CD4 cys diabody was successfully re-engineered by CDR-grafting (GK1.5 FR cDb) and aglycosylation (GK1.5 N80D cDb), resulting in a higher expression yield (~ tenfold increase) without impacting antigen specificity or affinity. Both cys-diabody variants were successfully ^89^Zr-radiolabeled with similar specific activity and radiochemical purity. ImmunoPET imaging of ^89^Zr-GK1.5 FR cDb and ^89^Zr-GK1.5 N80D cDb in immunocompetent mice showed CD4 antigen-specific lymphoid tissue uptake in vivo. ^89^Zr-GK1.5 FR cDb exhibited rapid hepatic clearance, resulting in significantly reduced uptake in lymph nodes and the spleen. Removal of the N-glycosylation motif in ^89^Zr-GK1.5 N80D cDb restored diabody-typical biodistribution (renal clearance), resulting in higher target tissue uptake.

**Conclusion:**

The novel reengineered anti-CD4 GK1.5 N80D cDb overcomes the previous production yield bottleneck and provides same-day ^89^Zr-immunoPET imaging for non-invasive, whole-body visualization of murine CD4^+^ T cells.

**Graphical Abstract:**

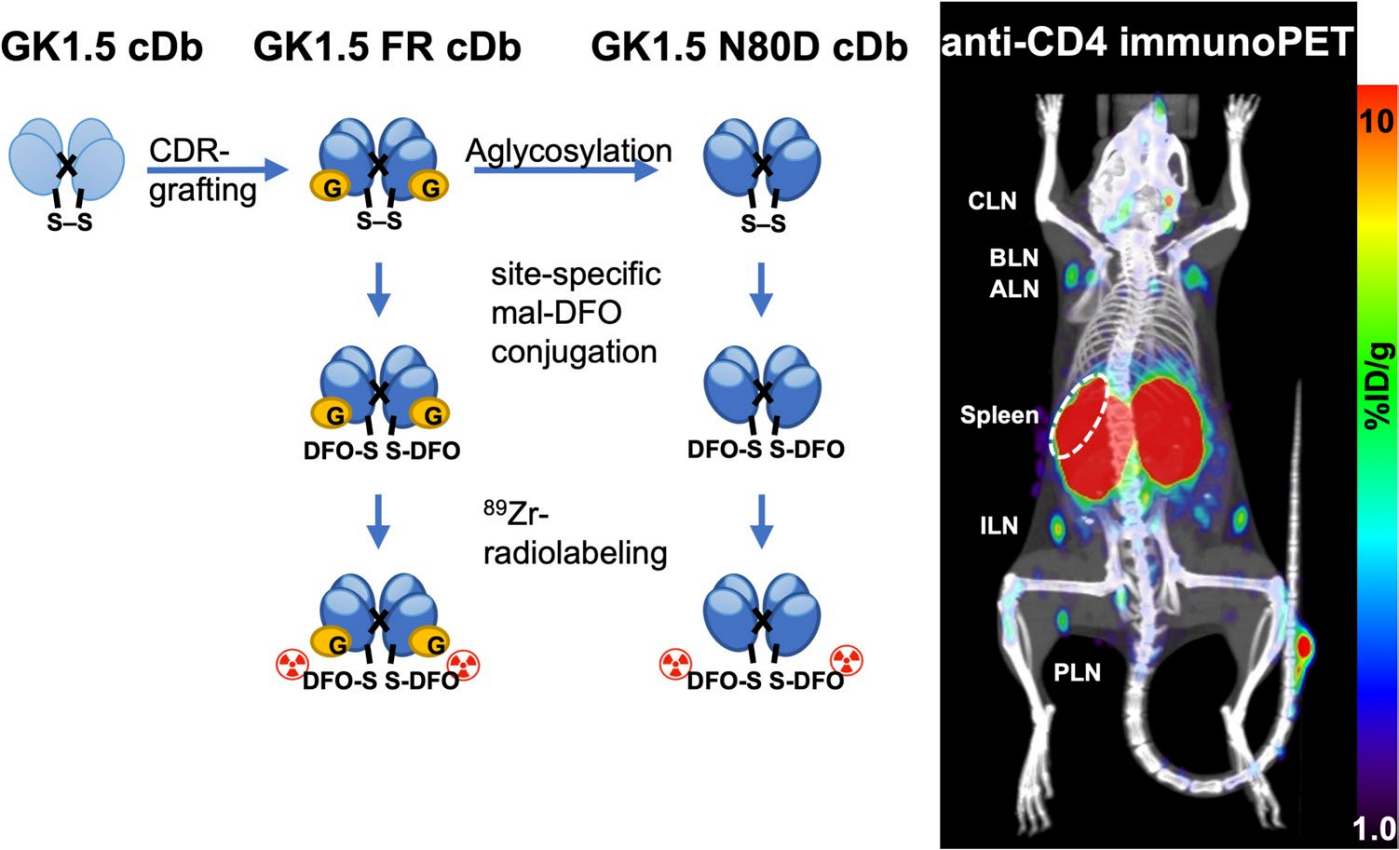

**Supplementary Information:**

The online version contains supplementary material available at 10.1007/s11307-025-02043-y.

## Introduction

T helper cells (CD4^+^ T cells) are crucial in regulating immunity; they ‘help’ activate phagocytes and B cells and expand and sustain cytotoxic CD8^+^ T cells. Other CD4^+^ T cell subsets, such as regulatory T cells (Tregs), suppress inflammation and immune responses. Therefore, CD4^+^ T cells are critical players in the pathology of infection, inflammatory and autoimmune diseases, and oncology [1]. The immunology of most autoimmune diseases (e.g., rheumatoid arthritis or inflammatory bowel disease, IBD) is complex and not very well understood, and better tools are needed to study the different immune cell subsets implicated in disease initiation and progression, such as CD4^+^ T cells [2, 3]. More recently, the role of tumor-infiltrating CD4^+^ T cells in the anti-tumor immune response, particularly in the context of immunotherapies such as immune checkpoint blockade (ICB) and adoptive cell transfer (ACT), has garnered increased interest [4, 5].

Monitoring immune cells through peripheral blood analysis is a minimally invasive process. Still, with the majority of CD4^+^ T cells residing in lymphoid tissues, this does not necessarily correlate to, nor is predictive for, lymphocytes infiltrating inflamed or cancerous tissue [6, 7]. Biopsies can provide more detailed information about T cells in the tissue of interest, but are invasive and suffer from heterogeneity within and between sample sites. Non-invasive whole-body positron emission tomography imaging using antibody fragments (immunoPET) can gain information on the number, location, and movement of highly dynamic immune cell populations [8]. Antibody fragments such as cys-diabodies (cDb, covalent scFv dimer) provide high specificity for cell surface-expressed antigens with rapid kinetics (molecular mass below renal clearance threshold: t_1/2_ 2–5 h) [9]. They can be combined with positron-emitting radionuclides to use the sensitivity and resolution of PET [10].

Previously, we engineered an anti-mouse CD4 cDb based on the GK1.5 hybridoma and used zirconium-89 immunoPET (^89^Zr-GK1.5 cDb) to demonstrate specific targeting and visualization of CD4^+^ T cells residing in lymphoid tissue of immunocompetent mice [11, 12]. Dose escalation studies determined that low-dose (2 µg) ^89^Zr-GK1.5 cDb resulted in high-contrast PET images at early time points. The antibody fragment caused minimal transient effects on proliferation, viability, and antigen modulation of CD4^+^ T cells (in contrast to the CD4^+^ T cell-depleting parental GK1.5 IgG) [13, 14]. Furthermore, we have successfully used this anti-CD4 immunoPET tracer to detect T cell repopulation in a model of hematopoietic stem cell transplantation [11] and to identify CD4^+^ T cells in the colon, ceca, and mesenteric lymph nodes of mice with DSS-induced colitis (a mouse model of inflammatory bowel disease) [15]. Despite these proof-of-concept studies, the further development of GK1.5 cDb for immunoPET imaging of CD4^+^ T cells in preclinical mouse models was impeded by its very low expression levels, making protein production ineffective (time, cost, effort).

In this work, we aim to re-engineer the GK1.5 cDb for higher expression to overcome the production bottleneck and, crucially, to retain CD4 antigen specificity, *in vivo* targeting capabilities, and biodistribution. The framework of a rat anti-mouse CD8 hybridoma (YTS169) [16], which we previously cloned and used to engineer antibody fragments (minibody, 169 Mb and cys-diabody 169 cDb) [17, 18], that showed high expression levels, was chosen as the acceptor framework for CDR grafting. The CDR-grafted cys-diabody (GK1.5 FR cDb) contains an N-glycosylation motif, which was removed to yield the aglycosylated derivative (GK1.5 N80D cDb). Both anti-CD4 cys-diabodies were evaluated for retained antigen binding and site-specifically radiolabeled with zirconium-89 (^89^Zr) for immunoPET imaging of *in vivo* CD4 + T cell targeting capacity and biodistribution.

## Materials and Methods

The supplementary information provides information regarding routine production, purification, and characterization of antibody fragments, as well as site-specific conjugation with DFO and ^89^Zr-radiolabeling**.**

### Reengineering of the Anti-CD4 Cys-diabody GK1.5

Parental anti-mouse CD4 antibody GK1.5 (rat IgG_2b,k_) was purchased (BioLegend Cat# 100401, RRID:AB_312686).

All six complementarity-determining regions (CDRs) of the anti-CD4 hybridoma GK1.5 were grafted onto the framework (VH and VL) of the YTS169 hybridoma (YTS169.4.2.1, rat anti-murine CD8), resulting in GK1.5 FR cDb [18]. Codon-optimized DNA was synthesized by GeneArt (Invitrogen), encoding for VH and VL connected by a five amino acid linker and including a C-terminal cysteine residue for site-specific conjugation, followed by a hexa-histidine tag (His6) for detection and purification (VH-G4S-VL-Cys-His). The construct was subcloned (via AgeI and NotI, New England Biolabs) into the mammalian expression vector pSecTag2A (Invitrogen). The glycosylation motif (N80-X-T, L81 in Kabat numbering) in the YTS169 VL framework was removed by site-directed mutagenesis, using forward primer 5'-GTG GAA GCC GAC GAT ACC GCC ACC TAC TAC-3'and reverse primer: 5'-GGC GGT ATC GTC GGC TTC CAC GGG GTC AAT-3'and the QuickChangeⓇ II XL Site-Directed Mutagenesis Kit (Agilent) according to the manufacturer's instructions, and resulting in GK1.5 N80D cDb. Both plasmids were transfected into Gibco 293-F cells (Thermo Fisher Scientific) using Lipofectamine 2000 (Invitrogen), and stable cell pools were selected using 300 µg/mL zeocin (InvivoGen).

### Isolation of Murine Splenocytes and Flow Cytometry

Site-specific conjugation was performed by mild reduction with a fivefold molar excess of TCEP (tris(2-carboxyethyl)phosphine, 30 min, room temperature, followed by incubation with a 20-fold molar excess of Alexa Fluor^TM^488 C5-maleimide (Invitrogen) overnight at 4 °C. Conjugated protein was purified using Pierce^TM^ Dye Removal Columns (Thermo Scientific).

Spleens were harvested and processed through a 40 µm cell strainer (Corning) in the presence of red blood cell lysis buffer. Splenocyte suspensions (1 × 10^6^ cells/100 µl, in PBS) were incubated with GK1.5 IgG-A488 or GK1.5 N80D cDb-A488 (2–10 µg) for 30 min on ice. Cells were analyzed by flow cytometry (LSR Fortessa X-20).

### ImmunoPET/CT

All procedures involving animals were performed under an approved UCLA Chancellor’s Animal Research Committee protocol. Groups of four mice (C57BL/6J, JAX 000664, female, 8–12 weeks) were injected via the tail vein with 2 µg of [^89^Zr]Zr-DFO-GK1.5 FR cDb (^89^Zr-GK1.5 FR cDb) or [^89^Zr]Zr-DFO-GK1.5 N80D cDb (^89^Zr-GK1.5 N80D cDb) (10–15 µCi or 0.4–0.6 MBq) in a total volume of 100 µL saline. For the CD4-blocking control group, mice were co-injected with 60 µg (3 mg/kg) non-radioactive GK1.5 N80D cDb. Mice were anesthetized with continuous 2% isoflurane, and 30-min dynamic scans followed by 10-min static scans at 1, 2, 4, and 22 h post-injection (p.i.) were acquired on an Inveon small animal PET scanner (Siemens). Each PET scan was followed by a 1-min CT acquisition (CrumpCAT; UCLA in-house small animal CT scanner). Images were reconstructed using the OSEM3D-MAP algorithm and are displayed as whole-body maximum intensity projections PET/CT overlays. Image analysis was performed using A Medical Imaging Data Examiner (AMIDE) [19].

### *Ex vivo* Biodistribution

Following the last imaging scan, mice were euthanized, blood was drawn by cardiac puncture, tissues were collected, and lymph nodes (inguinal, axillary, brachial, and cervical) were pooled. Tissues were weighed and gamma counted (Wizard 3″ 1480 Automatic Gamma Counter, Perkin Elmer), and the percent injected dose per gram of tissue (%ID/g) was calculated based on a standard containing 10% of the injected dose (decay-corrected).

### Statistical Analysis

Data are reported as Mean ± standard error of mean (SEM) unless stated otherwise. *Ex vivo* biodistributions are displayed as box-and-whisker plots (minimum to maximum). Statistical significance was determined using the Two-Way ANOVA model with a multiple comparisons correction test (Sidak, GraphPad Prism 9).

## Results

### Re-engineering of GK1.5 Cys-diabody

**Fig. 1:**
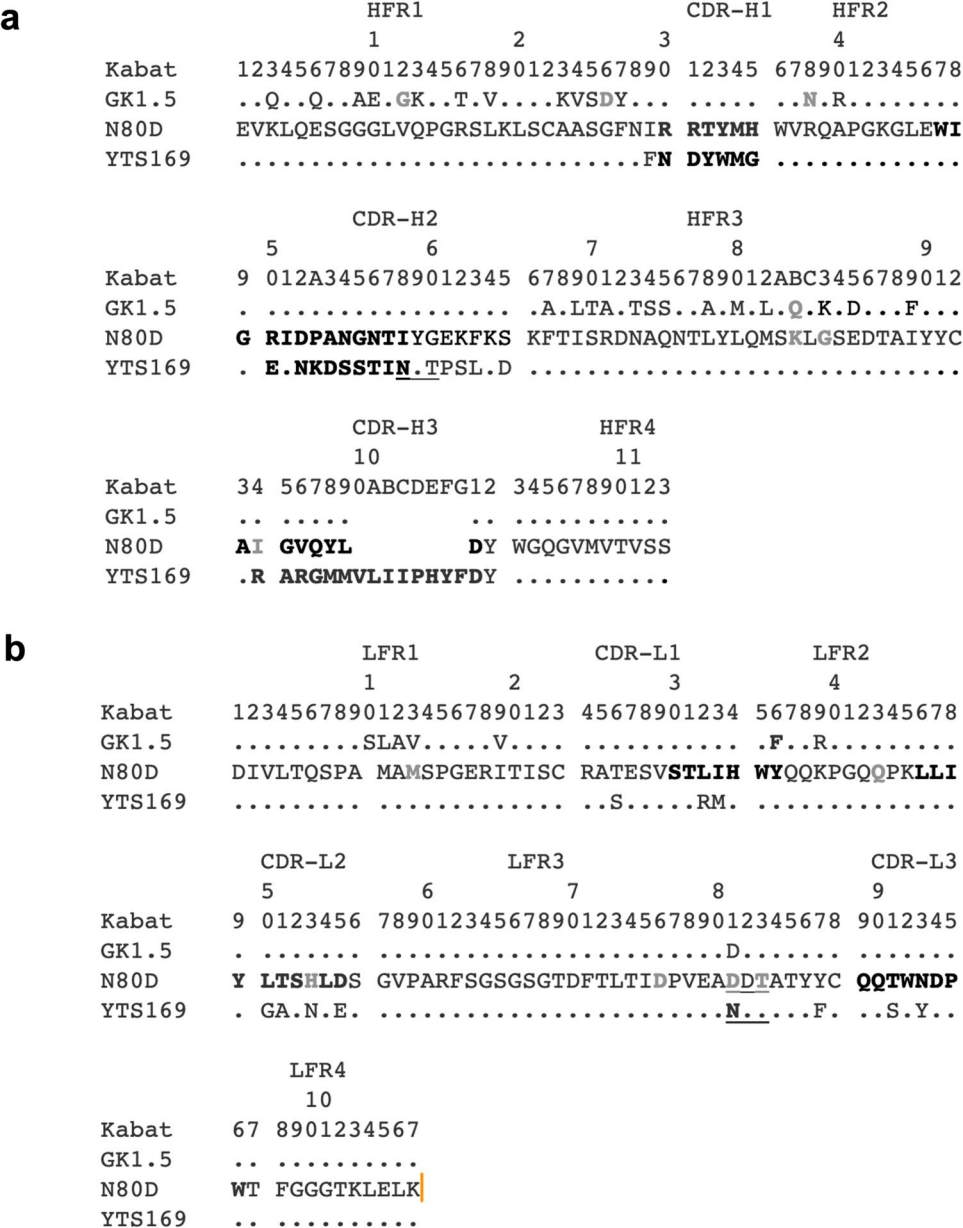
Sequence analysis and alignment of the variable domains of GK1.5, YTS169, and the novel GK1.5 N80D. **a**) Heavy chain variable domain. **b**) Light chain variable domain. Dots indicate identical residues, numbered according to the Kabat numbering scheme. Bold: contact definition, grey: unusual residues (< 1% of sequences), underlined: predicted N-glycosylation site

The anti-CD4 cys-diabody (GK1.5 cDb), based on the rat anti-murine CD4 hybridoma GK1.5, showed consistently low expression levels, severely limiting its development as a preclinical anti-CD4 PET tracer [11]. Therefore, GK1.5 cDb was re-engineered by grafting all six CDRs onto the homologous acceptor framework of the YTS169 hybridoma (rat anti-mouse CD8) with known higher expression **(**Fig. 1**)**. The CDR regions were defined according to the contact definition [20], and identical canonical CDR structures for donor and acceptor variable sequences were confirmed using abYsis (abysis.org, A fully integrated antibody discovery system, Version 3.4.1). The resulting anti-CD4 cys-diabody named GK1.5 FR cDb was produced in 293-F cells with yields of 4.6–5.6 mg/L supernatant (n = 2), about a 10- to 20-fold increase compared to the original GK1.5 cDb (0.36 mg/L supernatant) [11].

The YTS169 V_L_ framework contains an N-glycosylation motif (N-X-T) at residue 80 (Kabat numbering L81), and deglycosylation of GK1.5 FR cDb using PNGase F resulted in two additional bands of lower molecular weight visible in SDS-PAGE analysis, confirming that GK1.5 FR cDb is indeed glycosylated upon mammalian expression **(**Fig. 1, 2a**)**. Because glycosylation can influence the *in vivo* biodistribution and clearance of proteins, the asparagine residue at position 80 was changed to aspartic acid (N80D) by site-directed mutagenesis. The resulting GK1.5 N80D cDb migrated at a lower apparent molecular weight in SDS-PAGE analysis, corresponding to the theoretical molecular weight of the cDb (homodimer, M_W_ 52.2 kDa), and confirming successful genetic aglycosylation of the GK1.5 N80D cDb. The production yield for GK1.5 N80D cDb was 2.8 ± 0.6 mg/L (*n* = 4).

**Fig. 2:**
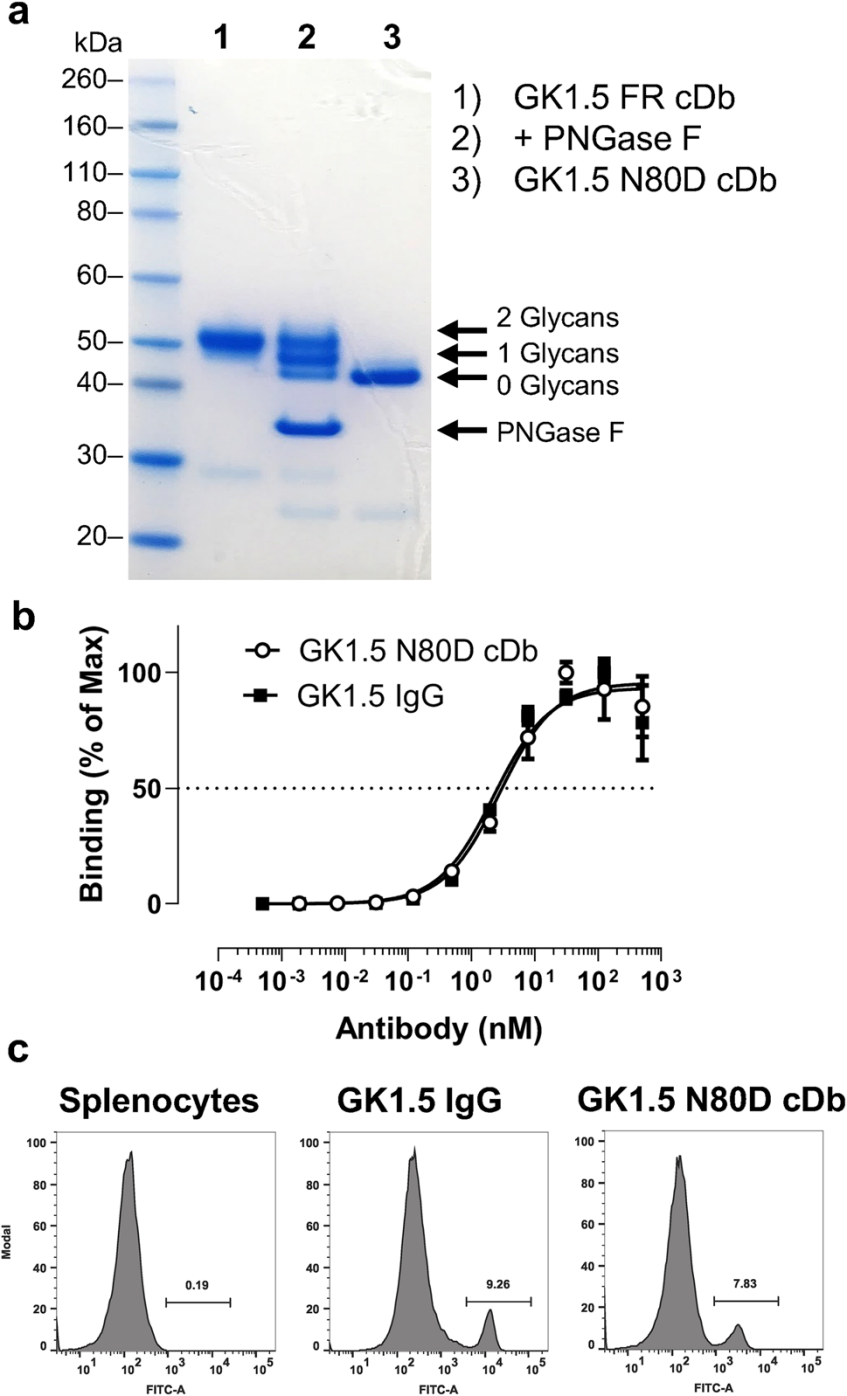
Biochemical characterization of GK1.5 FR cDb and GK1.5 N80D cDb. **a**) Deglycosylation. SDS-PAGE analysis of GK1.5 FR cDb (lane 1), deglycosylated GK1.5 FR cDb using PNGase F (lane 2), and genetically aglycosylated variant GK1.5 N80D cDb (lane 3). **b**) Binding to immobilized CD4 antigen (ELISA), shown is one of *n* ≥ 3 saturation binding curves to determine the half-maximal binding. **c**) Flow cytometry of mouse splenocytes stained with GK1.5 IgG-A488 or GK1.5 N80D cDb-A488

### Reengineered GK1.5 N80D cDb Retains Anti-CD4 Antigen Binding Affinity

Saturation binding curves (ELISA) on immobilized recombinant mouse CD4 showed that the CDR-grafted, genetically aglycosylated GK1.5 N80D cDb retains similar apparent affinity (EC_50_ 3.0 ± 1.0 nM, n = 4) compared to the parental full-length GK1.5 IgG (EC_50_ 2.25 ± 0.01 nM, n = 2) **(**representative binding curve shown in Fig. 2b**)**. No binding to control (BSA) was observed, confirming antigen specificity (data not shown). Specific binding of GK1.5 N80D cDb was further confirmed by incubation with soluble murine CD4 (molar ratio 1:2) and analysis of the antibody-antigen complexes by SEC (Fig. S1**)**.

Binding to mouse CD4 + T cells was tested using isolated splenocytes and GK1.5 N80D cDb site-specifically conjugated with fluorescent dye for flow cytometry (Fig. 2c, Fig. S2). GK1.5 N80D cDb-malAlexa488 stained a similar fraction of splenocytes (7.8%) as the parental GK1.5 IgG (9.3%), confirming retained cell binding specific for CD4^+^ T cells. Increasing the amount of GK1.5 N80D cDb from 2 µg to 5 and 10 µg did not further increase the fraction of stained cells, showing that binding was saturated (Fig. S3).

### Site-specific Conjugation of the Chelator mal-DFO to the Anti-CD4 cDbs

**Fig. 3:**
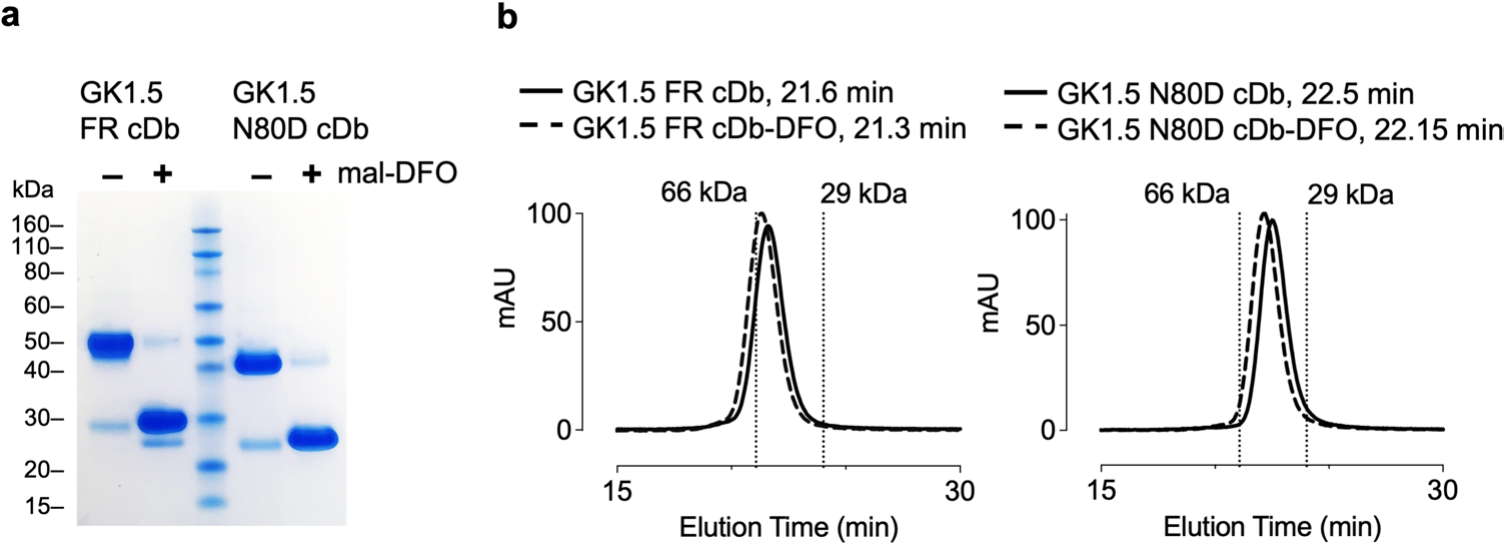
Site-specific DFO-conjugation. **a**) SDS-PAGE analysis of GK1.5 FR cDb and GK1.5 N80D cDb after reduction (TCEP) and conjugation with mal-DFO. **b**) Size exclusion chromatography comparing unconjugated and DFO-conjugated diabodies

For site-specific conjugation, the maleimide group of the bifunctional linker (mal-DFO) was reacted chemoselectively (pH 6.5–7.5) with thiols of the reduced anti-cD4 cDbs (C-terminal cysteine residues, Cys-tag). Successful conjugation of mal-DFO to GK1.5 FR cDb and GK1.5 N80D cDb was shown in SDS-PAGE analysis. The unconjugated anti-CD4 cDbs migrates with the apparent molecular weight of the covalent homodimer (M_W_ 52.2 kDa, Fig. 3a). Reduction with TCEP and conjugation of mal-DFO breaks the inter-chain disulfide bond and blocks reoxidation of the cys-tag, resulting in DFO-GK1.5 FR cDb and DFO-GK1.5 N80D cDb migrating at the size of the monomer (scFv, MW 26.1 kDa).

The DFO-conjugated anti-cD4 cDbs retained homodimeric diabody assembly as shown by size exclusion chromatography, where mal-DFO conjugated proteins eluted as single peaks slightly earlier (GK1.5 FR cDb-DFO: 21.3 min; GK1.5 N80D cDb-DFO: 22.15 min) compared with the unconjugated respective cDbs (GK1.5 FR cDb: 21.6 min; GK1.5 N80D cDb: 22.5 min) **(**Fig. 3b**).**

### ^89^Zr-immunoPET in Immunocompetent Mice (C57BL/6J)

**Fig. 4:**
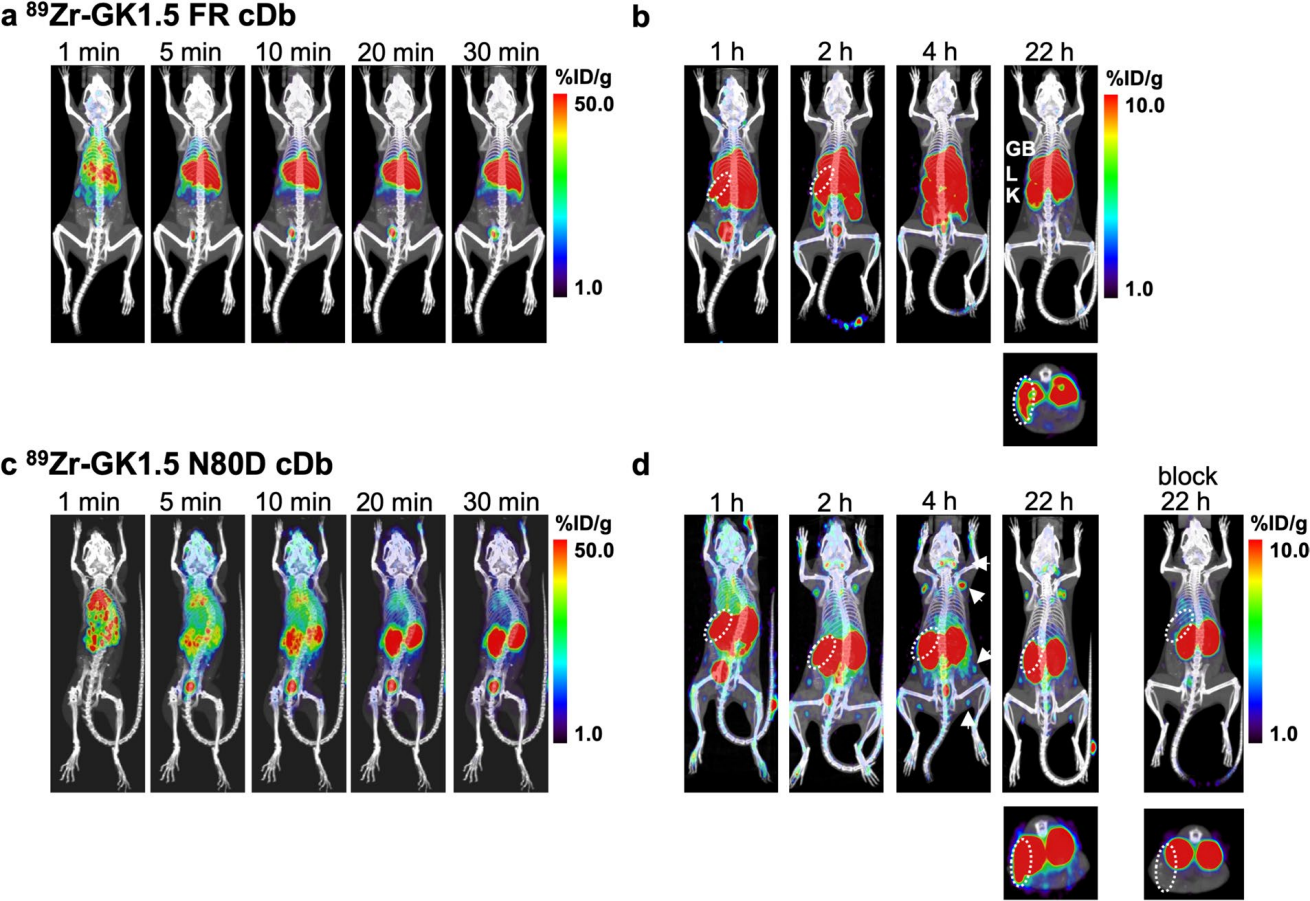
^89^Zr-anti-CD4 immunoPET imaging of immunocompetent mice (groups of *n* = 4). ^89^Zr-GK1.5 FR cDb (**a**, **b**) or ^89^Zr-GK1.5 N80D cDb (**c**, **d**) were injected via tail vein (2 µg/0.4–0.6 MBq). Mice underwent 30-min dynamic scans (a, c) followed by serial 10-min static scans at 1, 2, 4, and 22 h p.i. The blocking group was co-injected with non-radioactive GK1.5 N80D cDb at 3 mg/kg (60 µg). Each group's representative immunoPET/CT images are shown as whole-body MIP PET/CT overlay. For the 22 h p.i. timepoint, transverse Sects. (2 mm MIP) through the spleen are shown. Spleens are encircled (white dashed), *GB* gall bladder; *L* liver; *K* kidneys

Anti-CD4 cDb derivatives were successfully radiolabeled by chelation of ^89^Zr (results are summarized in Table 1) and yielded similar labeling efficiencies, specific activities, and radiochemical purities to the previously reported GK1.5 cDb [11, 13]. We have previously established that low-dose ^89^Zr-anti-CD4 cDb (2 µg) results in higher %ID/g in target tissues and reduced biological effects on CD4^+^ T cells compared to a higher protein dose (10–40 µg) [13]. Dynamic PET scans, taken up to 30 min post-tracer injection, clearly show the differential biodistribution and clearance of the two ^89^Zr-GK1.5 cDb derivatives. The glycosylated ^89^Zr-GK1.5 FR cDb exhibits rapid accumulation in the liver and gall bladder concomitant with rapid clearance from the blood (decreased blood pool signal in the heart) **(**Fig. 4a**)**. Targeting the CD4^+^ T cell in the spleen can be observed as early as 5 min p.i. Static scans at 1, 2, 4, and 22 h p.i. show activity in the intestines excreted with fecal matter, confirming hepatobiliary clearance **(**Fig. 4b**)**. Renal clearance and excretion into the urine (bladder) are also visible. Although specific uptake in the spleen is observed, it is lower compared to that of previously published parental ^89^Zr-GK1.5 cDb and is obstructed by activity in the liver and intestines [13, 15]. The very low uptake in lymph nodes is likely also a consequence of the tracers'rapid clearance.

**Table 1:** Radiolabeling of anti-CD4 GK1.5 cDb variants

	^89^Zr-GK1.5 FR cDb			^89^Zr-GK1.5 N80D cDb		
	Mean	SEM	N	Mean	SEM	N
Labeling efficiency (%)	99.7	nd	1	98.4	0.8	3
Specific activity (µCi/µg)	5.1	nd	1	6.6	0.8	3
Specific activity (MBq/µg)	0.19	nd	1	0.24	0.03	3
Radiochemical purity (%)	99.1	nd	1	99.8	0.1	3

In contrast, genetic aglycosylation restored diabody-typical biodistribution of ^89^Zr-GK1.5 N80D cDb, with longer retention of activity in the blood pool (heart) and clearance via the kidneys (Fig. 4c). Antigen-specific uptake in the spleen is observed starting around 5 min p.i., similar to other GK1.5 cDb variants. The longer plasma half-life of ^89^Zr-GK1.5 N80D cDb resulted in higher lymphoid tissue uptake. It peaked around 4 h p.i., most pronounced in the distinguishable lymph nodes (cervical, brachial, axillary, inguinal, and popliteal) (Fig. 4d**)**. Blocking with excess non-radioactive GK1.5 N80D cDb (3 mg/kg) confirmed the CD4-antigen specificity by decreasing the radioactive signal in lymphoid tissues (spleen and lymph nodes). At the same time, non-specific renal clearance remained visible (Fig. 4d, right panel).

### *Ex vivo* Biodistribution

**Fig. 5:**
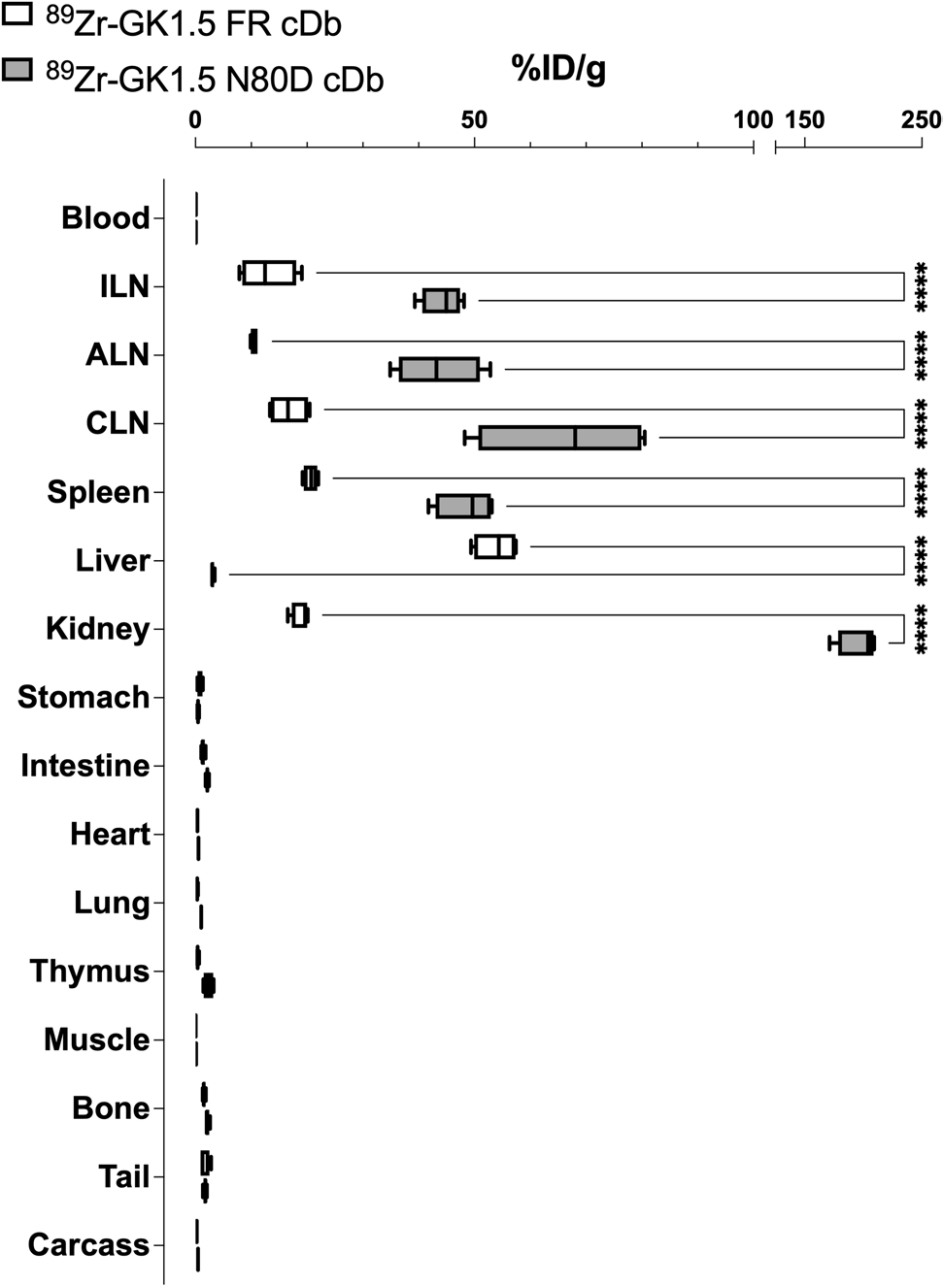
*Ex vivo* biodistribution of ^89^Zr-GK1.5 FR cDb and.^89^Zr-GK1.5 N80D cDb (22 h p.i.), *n* = 4 per group. Tissues of interest were harvested, weighed, and gamma counted to calculate the %ID/g based on decay-corrected standards. Values are depicted as box-and-whiskers. Two-way ANOVA (alpha threshold 0.05), Sidak's multiple comparison test, with individual variances computed for each comparison; 0.1234 (ns), 0.0332 (*), 0.0021 (**), 0.0002 (***), < 0.0001 (****)

Following the final PET scan (22 h p.i.), *ex vivo* biodistribution studies were conducted to determine the %ID/g values in respective tissues **(**Fig. 5**, **Table 2**)**. For the glycosylated ^89^Zr-GK1.5 FR cDb, uptake in the spleen (20.7 ± 0.6%ID/g) and lymph nodes (10.5–16.7%ID/g) confirmed specific targeting to CD4 + T cell-containing tissues. The tissue with the highest non-specific tracer retention was the liver (54 ± 2%ID/g), followed by the kidneys (19 ± 1%ID/g), confirming the observations made from the immunoPET images, which showed primarily hepatobiliary clearance and some secondary renal clearance. In comparison, the specific uptake of ^89^Zr-GK1.5 N80D cDb in the spleen (49 ± 3%ID/g) and lymph nodes (44–66%ID/g) was demonstrably superior, resulting in a more than doubled spleen-to-blood ratio of 485 (207 for ^89^Zr-GK1.5 FR cDb). In the group of mice co-injected with non-radioactive GK1.5 N80D cDb, uptake of ^89^Zr-GK1.5 N80D cDb was decreased to 8.1 ± 0.4%ID/g in the spleen and 10–12.5%ID/g in the lymph nodes. Renal clearance of ^89^Zr-GK1.5 N80D cDb was confirmed by high activity retention in the kidneys (> 200%ID/g).

**Table 2:** *Ex vivo* biodistribution of ^89^Zr-GK1.5 FR cDb and ^89^Zr-GK1.5 N80D cDb. 22 h p.i., values are shown as %ID/g mean ± SEM, *N* = 4

	^89^Zr-GK1.5 FR cDb		^89^Zr-GK1.5 N80D cDb		^89^Zr-GK1.5 N80D cDb blocked	
	Mean	SEM	Mean	SEM	Mean	SEM
Blood	0.1	0.007	0.1	0.005	0.4	0.1
Inguinal LN	13.0	2.5	44.3	1.9	18.3	5.5
Axillary, brachial LN	10.5	0.3	43.5	3.8	16.6	5.0
Cervical LN	16.7	1.8	66.2	7.8	20.0	4.7
Spleen	20.7	0.6	48.5	2.7	9.7	1.3
Liver	53.9	2.0	3.1	0.1	5.3	1.0
Kidneys	19.0	0.8	197.1	8.8	305.3	20.3
Stomach	0.8	0.03	0.5	0.1	0.4	0.1
Intestines	1.3	0.1	1.0	0.2	1.3	0.2
Heart	0.4	0.1	2.3	0.02	0.9	0.2
Lung	0.3	0.02	0.1	0.1	5.7	4.6
Thymus	0.4	0.1	2.2	0.5	1.9	0.4
Muscle	0.1	0.4	1.8	0.02	0.3	0.1
Bone	1.6	0.01	0.5	0.2	1.7	0.4
Tail	1.6	0.03	0.5	0.2	6.4	1.5
Carcass	0.3	0.1	1.0	0.01	0.6	0.1
	Mean	95% CI	Mean	95% CI	Mean	95% CI
Spleen-to-blood ratio	207	174–253	485	403–582	24.3	13.0–64.5

## Discussion

Non-invasive imaging of CD4^+^ T lymphocytes holds great promise for enhancing our understanding of the critical role this immune cell subset plays in disease (e.g., HIV/AIDS, inflammatory diseases) or in response to therapy, such as vaccine development, transplantation/graft-versus-host disease, and immunotherapy for cancer. The increased interest in visualizing the spatiotemporal *in vivo* biodistribution of T cells, including trafficking, persistence, or expansion at tumor sites and activation or exhaustion phenotype, is reflected in the plethora of imaging modalities and targets being explored in preclinical and clinical trials, as reviewed in [21]. A humanized antibody fragment targeting human CD4^+^ T cells (IAB41 minibody, ImaginAb) is currently being explored for imaging in humanized mice and clinical translation [22, 23]. Another approach utilizes anti-human CD4 single-domain antibodies and shows the accumulation of ^64^Cu-radiolabeled CD4-Nb1 in lymphoid tissues of a human CD4 knock-in mouse model [24]. However, for preclinical cancer immunology research and evaluation of response to immunotherapies, syngeneic tumors transplanted into immunocompetent mice remain readily available, reproducible, and widely used models that require mouse-specific imaging tracers [25, 26].

Previous proof-of-principle studies using the anti-CD4 cys-diabody GK1.5 cDb for ^89^Zr-immunoPET imaging confirmed the ability to detect CD4 + T cells in the lymphoid tissues (spleen and lymph nodes) of immunocompetent mice, without significant impact on the function or proliferation of the target cells [13]. Furthermore, ^89^Zr-GK1.5 cDb was successfully used to visualize the reconstitution of CD4^+^ T cells after hematopoietic stem cell transplantation and in a model of inflammatory bowel disease [11, 13, 15]. Wider use of anti-CD4 immunoPET imaging could be facilitated by overcoming the very low expression levels of the GK1.5 cDb.

GK1.5 cDb was originally cloned based on the genetic information encoding the variable domains obtained from a hybridoma (ATCC TIB-207, GK1.5, rat IgG2b,_K_) and assembled into a functional cys-diabody (dimer of scFv). However, the use of degenerate primers can introduce mutations at the start and end of the sequence, which might impact expression and folding and result in low protein yields [27]. While CDR-grafting is more commonly used to humanize antibodies [28], we hypothesized that transplanting the antigen binding site onto a rodent framework with known higher expression levels could overcome the unsatisfactory protein expression yields of GK1.5 cDb.

The six CDR loops of GK1.5, based on the contact definition (i.e., residues that take part in interactions with antigen and are part of the canonical sequence for loop structure), were grafted onto the variable domain framework of the rat anti-CD8 hybridoma YTS169 [11, 20]. CDR-grafting resulted in a rodent cys-diabody (GK1.5 FR cDb) with retained specificity and affinity for murine CD4^+^ T cells and significantly improved expression yields.

One unusual sequence feature was identified in the variable light chain domain framework (residue 80, Kabat L81N), constituting a potential N-glycosylation site. Deglycosylation using PNGase F confirmed that the recombinant protein, produced in a mammalian expression system, is indeed glycosylated. *N*-linked glycans in the variable domains, so-called Fab glycans, are a product of somatic hypermutation and antibody diversification. Fab *N*-glycosylation function and effects are less understood than Fc-glycosylation, but variable domain glycans are reported to impact antigen–antibody binding, specificity, affinity, and antibody stability [29–32]. Furthermore, glycan-binding receptors in the liver can also significantly affect blood clearance and catabolism of glycoproteins [33, 34]. The glycosylated GK1.5 FR cDb exhibited unexpectedly rapid clearance from the blood and accumulation in the liver that is nontypical for a 50 kDa cys-diabody. Investigating the Fab glycan composition and the impact of specific liver glycan receptors on the clearance exceeded the scope of this study, but could inform future antibody modification. Genetic aglycosylation resulted in the variant GK1.5 N80D cDb and restored renal clearance and plasma half-life similar to that reported for other diabodies (2–5 h) [35–37]. The [^89^Zr]Zr-DFO is site-specifically conjugated to the C-terminal cysteine, which is likely reabsorbed in the kidney’s proximal tubules, leading to retention of the residualizing radiometal. These results corroborate the previous observation that the anti-CD8 minibody ([^64^Cu]Cu-NOTA-YTS169 Mb), which contains the N-glycosylation, exhibited rapid clearance from the blood. At the same time, the aglycosylated diabody ([^89^Zr]Zr-malDFO-169 N85D cDb) showed renal clearance at the expected rate [17, 18].

The novel CDR-grafted anti-CD4 cDb variants retained low-nanomolar affinity (3.0 ± 1.0 nM) comparable to the previously published GK1.5 cDb (2.7 ± 0.2 nM) and the parental GK1.5 IgG (1.1 ± 0.06 nM) [13]. Furthermore, flow cytometry analysis of mouse splenocytes showed that GK1.5 N80D cDb stained the same fraction of CD4^+^ T cells as the parental GK1.5 IgG. Importantly, these findings confirmed that CDR grafting did not affect affinity and specificity.

Monitoring CD4^+^ T cells over time would benefit from the capability to conduct serial immunoPET imaging in the same animal. We have not tested the immunogenicity of the rat variable domain framework in mice or its impact on the CD4 T cell population; however, further ‘’murinization” may be required. Another limitation of the anti-CD4 cys-diabody is its inability to distinguish between CD4^+^ T cell subsets (e.g., immunosuppressive Tregs or proinflammatory Th1 T cells) and other immune cell subsets that can express CD4, such as dendritic cells [38]. Combinations of distinct biomarkers could be explored for a more comprehensive *in vivo* phenotyping of the immune tumor microenvironment.

The ^89^Zr-labeled GK1.5 N80D cDb, with its rapid blood clearance at low protein doses, yielded high-contrast PET images as early as 2–4 h p.i. In this model (non-tumor bearing C57BL/6J), ^89^Zr-GK1.5 N80D cDb reached higher uptake in lymphoid tissues than previously reported for the mCD4-Mb minibody (IAB46M2-18) [26]. The low background in normal tissues, except for the kidney (organ of clearance), could facilitate the detection of tumor-infiltrating CD4 + T lymphocytes in tissues that often exhibit a higher background, such as the liver. Furthermore, the cys-diabody format would be suitable for radiolabeling with shorter-lived radionuclides, such as fluorine-18 (^18^F). We have previously shown that cys-diabodies can be site-specifically or randomly ^18^F-radiolabeled for same-day imaging (4 to 8 h p.i.) without impacting their biodistribution or pharmacokinetics [39, 40]. An ^18^F-labeled anti-CD4 cDb would further reduce the absorbed radiation dose, which could be crucial for serial imaging of radiosensitive CD4^+^ T cells [41]. Quantitative image analysis could provide a more comprehensive profile of the pharmacokinetics and biodistribution of immunoPET tracers, especially at earlier time points. However, limitations such as partial volume effects and the proximity of tissues with high signal retention need to be considered[42]. The integration of artificial intelligence and deep learning for PET image reconstruction and analysis holds promise for significant improvements[43].

In this study, we have re-engineered an anti-mouse CD4-specific cys-diabody (GK1.5 N80D cDb) that can be produced with sufficient yields for imaging studies in murine models of disease and response to therapy, enabling rapid and non-invasive monitoring and quantification of CD4 + T Cells. The cys-diabody format allows site-specific radiolabeling, provides a good compromise between rapid clearance, effective targeting, and reduced radiation dose, and is biologically inert (no Fc region). ^89^Zr-GK1.5 N80D cDb immunoPET imaging in preclinical disease models has the potential to guide the development and translation of anti-CD4 immunoPET imaging and other CD4-targeted immunotherapies.

## Conclusions

The re-engineered anti-CD4 cys-diabody enables non-invasive, whole-body immunoPET imaging of the CD4^+^ T cell compartment in preclinical mouse models. The ability to spatiotemporally monitor CD4 + T cells both locally (at the disease site) and systemically (in lymph nodes and the spleen) can further our understanding of their role in disease and response to therapy, as well as their value as predictive or prognostic markers.

## Supplementary Information

Below is the link to the electronic supplementary material.

Supplementary file1 (DOCX 1101 KB)

## Data Availability

All data displayed or supporting the findings of this study are available from the corresponding author upon request.
