# Supplementary material for: Reengineered Anti-CD4 Cys-diabody Variants for 89Zr-immunoPET of CD4+ T Cells in Immunocompetent Mice
Source: Mol Imaging Biol. 2025 Aug 7;27(5):774–84. doi: 10.1007/s11307-025-02043-y (PMC12628392; doi:10.1007/s11307-025-02043-y)
Supplement: Supplementary file 1 — Supplementary file1 (DOCX 1101 KB) [file 11307_2025_2043_MOESM1_ESM.docx]

Supplemental data for:

Reengineered anti-CD4 cys-diabody variants for ^89^Zr-immunoPET of CD4^+^ T cells in immunocompetent mice

**Felix B Salazar^1,2^, Richard Tavaré^1,3^, Arya Ökten^1,4^, Maciej Kujawski^2^, Anna M Wu^1,2^ and Kirstin A Zettlitz^1,2*^**

^1^Crump Institute for Molecular Imaging, Department of Molecular and Medical Pharmacology, David Geffen School of Medicine at University of California Los Angeles, Los Angeles, CA, USA

^2^Department of Immunology and Theranostics, Arthur Riggs Diabetes and Metabolism Research Institute, Beckman Research Institute of the City of Hope, Duarte, CA, USA

^3^Current Address: Regeneron Pharmaceuticals, Inc., Tarrytown, NY, USA

^4^Current Address: Department of Immunobiology, Yale University, New Haven, CT, USA

***Correspondence:**Kirstin A Zettlitz
[kzettlitz@coh.org](mailto:kzettlitz@coh.org)

**Materials and Methods**

## Production of anti-CD4 cys-diabodies

Cells were grown in TripleFlasks (Nunc) for static culture protein production and allowed to reach 70-80% confluence. The growth medium was replaced with Opti-MEM (reduced serum medium, Gibco), and the supernatant was collected every 3-4 days for up to three weeks. Pooled supernatants were concentrated using Pellicon®XL50 with Biomax® 10 kDa Membrane in the Labscale™ TFF system (Millipore), and recombinant protein was purified by immobilized metal affinity chromatography (IMAC) using HisTrap™ HP 1 ml column in an Äkta purifier (both GE Healthcare). Briefly, concentrated supernatant (20 mM imidazole added) was loaded onto the HisTrap column equilibrated with wash buffer (50 mM sodium phosphate buffer, 250 mM NaCl, 20 mM imidazole, pH 8.0). Bound protein was eluted by a gradual increase of elution buffer (50 mM sodium phosphate buffer, 250 mM NaCl, 500 mM imidazole, pH 8.0). Purified protein-containing fractions were pooled and dialyzed twice against 1xPBS. The purity and integrity of recombinant protein were confirmed by SDS-PAGE analysis (Coomassie stained) and size exclusion chromatography.

## Size exclusion chromatography

Purified proteins were analyzed using a Superdex-75 HR10/30 column in an Äkta purifier (GE Healthcare) with PBS as mobile phase at a flow rate of 0.5 ml/min. The following standard proteins were used: BSA (66 kDa) and carbonic anhydrase (29 kDa) (Sigma-Aldrich).

## Deglycosylation

Cys-diabodies (10 µg) were incubated with 1 µL PNGase F (New England BioLabs) in 10 µL volume at 37°C for 24 h. Untreated and deglycosylated proteins were denatured (70°C, 10 min), analyzed by SDS-PAGE (2 µg/lane), and stained with Coomassie.

## ELISA

Recombinant soluble mCD4 antigen production was previously described [1]. Antigen was coated onto Costar™ 96-well EIA/RIA plates (Corning) at a 5 µg/mL concentration in 0.1 M sodium carbonate bicarbonate buffer, pH 9.6, at 4°C, overnight. All binding and washing steps were conducted at room temperature. 1% BSA in sodium carbonate bicarbonate buffer, pH 9.6, was used to block the remaining binding sites (2 h). Biotinylated (biotin-maleimide, Sigma-Aldrich) primary antibodies (cys-diabodies, parental GK1.5) were diluted in blocking buffer at the indicated concentrations in triplicate (serial dilution, 500 nM – 0.052 pM) and incubated for 1 h. Bound antibodies were detected using Alkaline Phosphatase Streptavidin (Jackson ImmunoResearch Laboratories Inc), developed with *p*-Nitrophenyl Phosphate (Sigma-Aldrich) and read at 405 nm in a microplate reader (Infinite® 200 Pro, Tecan). Saturation binding curves were fitted to a one-site specific binding model (GraphPad Prism). The apparent affinity (half-maximal binding) was calculated from three independent experiments.

## Site-specific conjugation with mal-DFO

The cys-diabodies in PBS (approximately 100 µg at 1.4-2.4 mg/mL) were reduced under mild conditions: TCEP (tris(2-carboxyethyl)phosphine), 5-fold molar excess, 30 min, room temperature. A 20-fold molar excess of deferoxamine-maleimide (mal-DFO, Macrocyclics, B-772) was added to the reduced protein and incubated at 4°C overnight. Excess mal-DFO was removed using size exclusion chromatography spin columns pre-blocked with 1% FBS, PBS, and equilibrated with PBS (Micro Bio-Spin™ 6 Columns, Bio-Rad).

## ^89^Zr-radiolabeling

[^89^Zr]Zr-oxalate (3D Imaging) was neutralized (0.45-volume of 2 M Na_2_CO_3_, 2.5-volume of 1 M HEPES) and incubated with DFO-conjugated cDb (0.167-0.281 MBq/µg or 4.5-7.6 µCi/µg) in 1 M HEPES (pH 7.0) following the protocol by Vosjan et al., 2010 [2]. Removal of free ^89^Zr and buffer exchange was performed as described above, and size exclusion chromatography spin columns were equilibrated with 1% FBS in PBS to reduce nonspecific binding. Labeling efficiency and radiochemical purity were determined using instant thin-layer chromatography strips for monoclonal antibody preparation (ITLC, Biodex Medical Systems) with 20 mM citric acid (pH 5.0) as the mobile phase.

**Supplemental Figures**


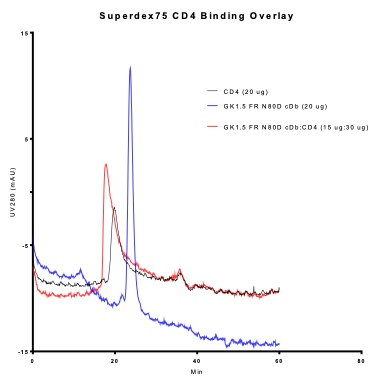


**Figure S1. Antigen binding in solution.**

Size exclusion chromatography of soluble murine CD4 antigen (black), GK1.5 N80D cDb (blue), and CD4:GK1.5 N80D cDb 2:1 complex (red) after incubation in solition.


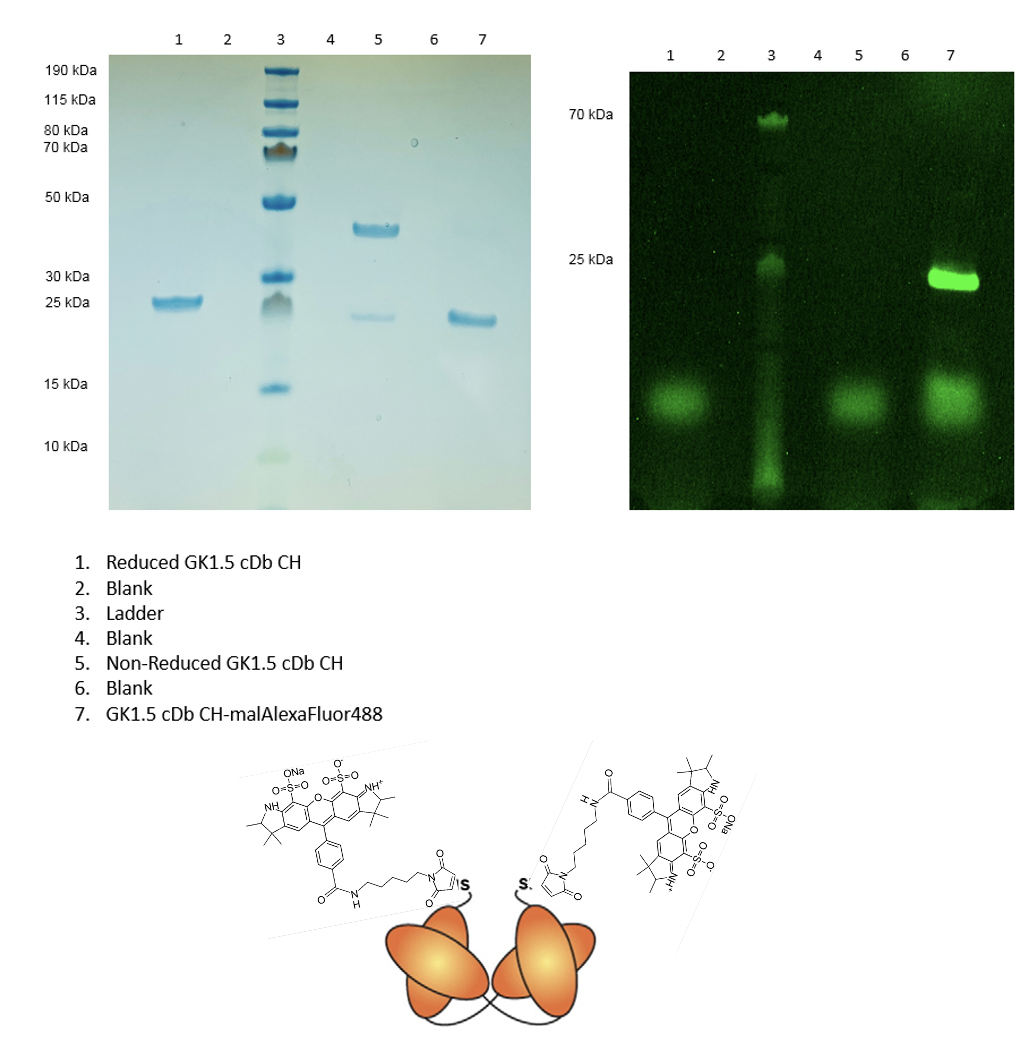


**Figure S2. Site-specific conjugation of GK1.5 N80D cDb with Alexa Fluor^TM^ 488 C5 Maleimide.** GK1.5 N80D cDb was site-specifically conjugated after mild reduction (5-fold TCEP) by incubating with 10-fold Alexa Fluor^TM^ 488 C5 Maleimide. Conjugation with the fluorophore prevents re-oxidation of the disulfide bond, and GK1.5 N80D cDb-A488 migrates at the apparent molecular weight of the monomeric scFv (ca. 26 kDa) concurrent with the fluorescent signal. Unconjugated GK1.5 N80D cDb was analyzed as a control under reducing and non-reducing conditions.


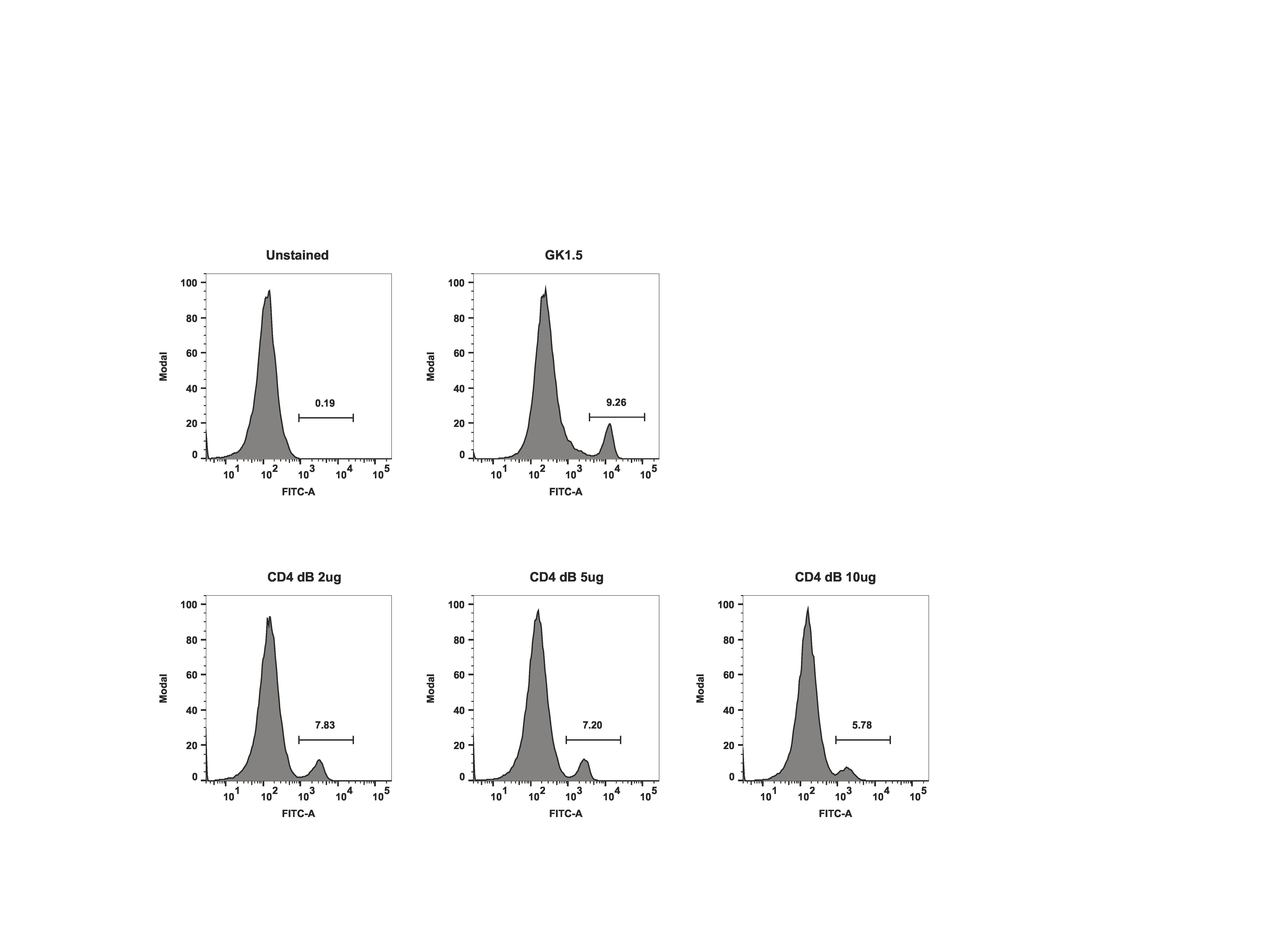

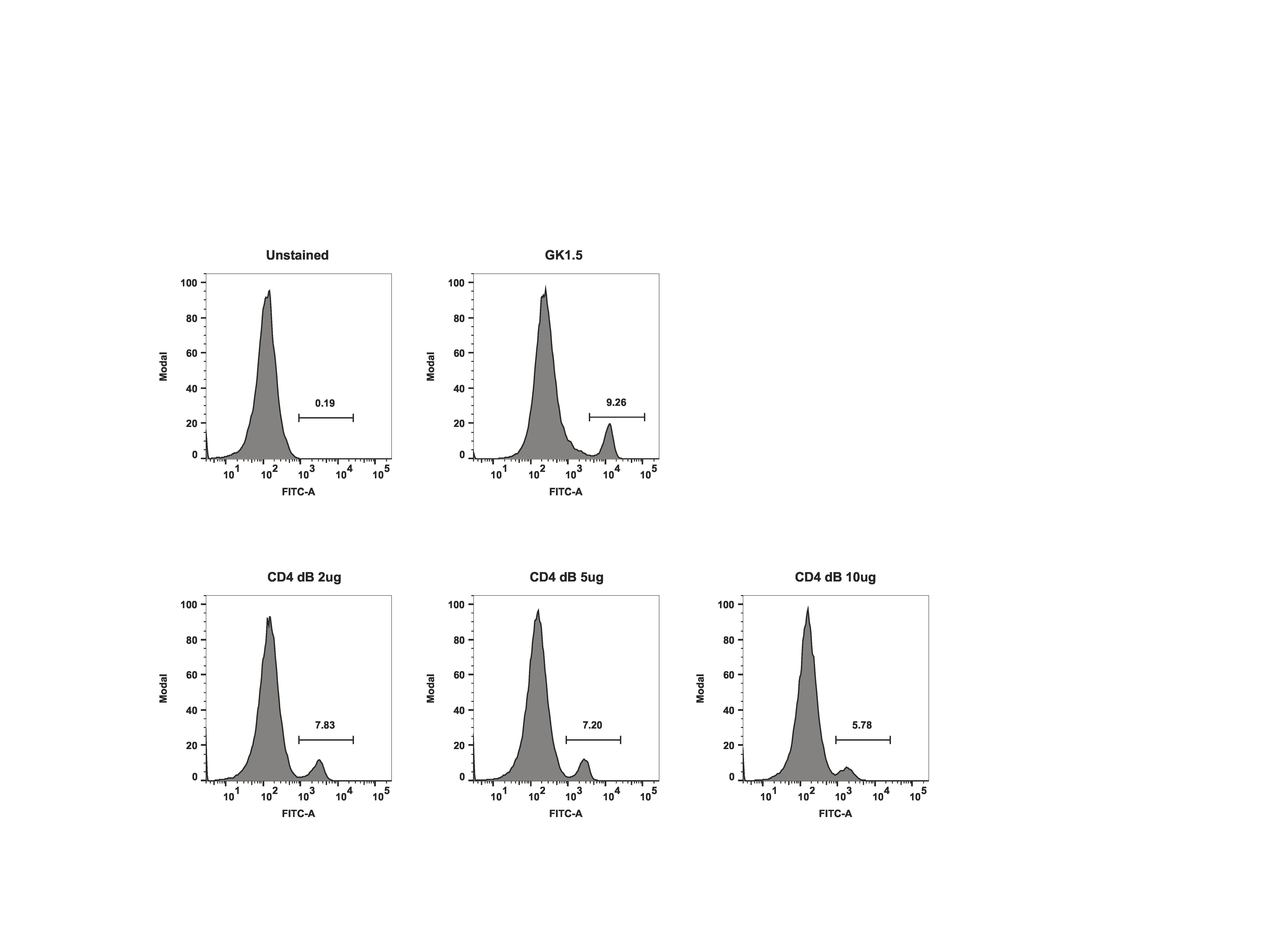


Splenocytes

GK1.5 IgG

GK1.5 N80D cDb, 2 µg

5 µg

10 µg

**Figure S3. Flow cytometry of splenocytes.**  Single-cell suspensions were prepared from the spleens of C57BL6/J mice and incubated with full-length anti-CD4 GK1.5 IgG or increasing doses of Gk1.5 N80D cDb-A488.

# Bibliography

1. Freise AC, Zettlitz KA, Salazar FB, Lu X, Tavare R, Wu AM (2017) ImmunoPET Imaging of Murine CD4(+) T Cells Using Anti-CD4 Cys-Diabody: Effects of Protein Dose on T Cell Function and Imaging. Mol Imaging Biol 19:599-609.

2. Vosjan MJ, Perk LR, Visser GW, et al. (2010) Conjugation and radiolabeling of monoclonal antibodies with zirconium-89 for PET imaging using the bifunctional chelate p-isothiocyanatobenzyl-desferrioxamine. Nat Protoc 5:739-743.
